# Long-Term Evaluation of a UK Community Pharmacy-Based Weight Management Service

**DOI:** 10.3390/pharmacy8010022

**Published:** 2020-02-19

**Authors:** Gareth Evans, David Wright

**Affiliations:** 1 Community Pharmacist at Waistaway Ltd. 26 Seathwaite, Huntingdon PE29 6UY, UK; 2 School of Pharmacy, University of East Anglia, Norfolk NR4 7TJ, UK; D.J.Wright@uea.ac.uk

**Keywords:** weight management, community pharmacy, very low calorie diet, Lipotrim

## Abstract

Obesity increases the risk of cardiovascular disease, type 2 diabetes and cancer, reducing both the quality and quantity of life. Consequently, government healthcare costs are significant. A greater than 5% reduction in weight has been shown to result in significant improvements in type II diabetes, blood pressure and cholesterol levels and therefore effective interventions are required. This paper reports the results from 17 years of delivering a private, individualised very low calorie diet (VLCD) programme in community pharmacy. In line with national guidelines, a community pharmacy-based private weight management service was set up to support individuals over the age of 18. After assessment for clinical suitability, individuals were offered either a flexible weight loss plan or a strict weight loss plan using a very low calorie diet (VLCD). The VLCD was delivered using the protocols of the proprietary programme, Lipotrim™. These individuals followed one or more dieting sequences, defined as at least one week of attendance whilst following the VLCD, without discontinuation, producing at least a start and end weight. Data were recorded weekly and audited for this report including weight and BMI on initial presentation, weight and BMI lost and % weight and BMI loss. A total of 1875 dieting sequences were recorded from 1023 dieters. In 1261 (67.3%) sequences, a medically beneficial weight loss of >5% was achieved. Overall, the cohort demonstrated mean (sd) % weight losses of 10.1% (7.7). Mean (sd) % weight losses seen in people with type 2 diabetes was 10.4% (2.7) and 10.6% (5.9) in hypertension. In total, 555 diet sequences accessed long-term weight maintenance support. In 173 (31%) of these cases, a second weight check post weight loss could not be made. The remaining 382 individuals presenting showed a mean (sd) weight gain of only 1.4 kg (4.3) equating to a mean (sd) % weight gain of only 1.8% (4.6) over a mean (sd) number of days post weight-loss of 132 days (179). The results from this long-term review demonstrate that with proper provision of a nutritionally complete VLCD, through private service provision, community pharmacies can make a significant contribution to reducing the obesity epidemic at no cost to state-funded health systems.

## 1. Introduction

Obesity is a major cause of type 2 diabetes, cardiovascular disease, hypertension, stroke, and certain types of cancer, with reduced quality of life and premature death a frequent outcome [1]. With 28.7% of adults classified as obese and a further 35.6% overweight in England [2], this creates a significant but preventable demand on the National Health Service (NHS), who provide free healthcare to all UK citizens at the point of delivery. Community pharmacy offers services through NHS contracts but there is flexibility to provide additional private services. The ‘Globesity’, as described by the World Health Organisation, is not limited to a small number or countries but to many of those across the globe [3]. Weight loss of greater than 5% is generally recognised as the clinically beneficial threshold for reducing type 2 diabetes incidence and consequences, improving both systolic and diastolic blood pressures, HDL cholesterol and triglyceride levels [4]. Safe and effective interventions are required to achieve this [5].

A recent systematic review comparing different approaches to weight management found that weight loss was greatest with meal replacements, closely followed by pharmacotherapy. Whilst high protein diets had a small but significant effect, exercise and dietary supplements were found to provide no benefit [6].

Historically, many pharmacotherapies for weight loss have been withdrawn due to side effects. A new therapy, Liraglutide, reported greater than 5% of weight loss in 63.2% of participants compared to 27.1% with placebo and this difference was significant [7]. It was seen that 33.1% with Liraglutide and 10.6% with placebo lost more than 10% of their body weight [7]. Interestingly, the placebo group performed better than lifestyle programs [8]. Unfortunately, this therapy requires daily injections, is only available on the NHS as Victoza (Saxenda currently unavailable on the NHS) for use within individuals with diabetes and consequently it has limited reach and long-term acceptability.

The average weight losses of slimming club services are insufficient to provide clinical benefit with individuals accessing Weight Watchers™ reported to lose on average 4.2 kg ± 4.1 (SD) at 3 months, reducing to 3.7 kg ± 6.4 (SD) at 12 months. Whilst Weight Watchers was found to be non-inferior to other commercial programs, it was still found to be superior to an equivalent NHS group based programme [8].

A systematic review of the evidence for low calorie diets or VLCD found that at 2 years Weight Watchers reported an average of 3.2% reduction of weight, but those medically supervised on a VLCD could achieve between 15% and 25% reductions [9].

Bariatric surgery where the stomach is operated upon to produce weight loss [10,11] has been shown to be effective. With significant reported average weight losses variable depending on choice of surgery, this approach is often used to reduce diabetes incidence [12], hypertension, heart failure and mortality [13]. The use of invasive surgery, including procedures such as using lap-bands, to expedite weight loss is however a last choice treatment and only for severe obesity.

Within the UK, the National Institute for Healthcare Excellence separates possible obesity interventions into 4 Tiers, ranging from basic health-promotion activity at Tier 1 through to bariatric surgery at Tier 4 [14,15]. The mainstay of current advice, lifestyle intervention at Tiers 1 and 2, whether through slimming clubs or structured NHS services, offer only modest weight losses.

Due to extended opening hours, privacy, confidentiality and open access to a healthcare professional, community pharmacies are increasingly being used to effectively promote healthy lifestyles [16,17,18]. In contrast, a recent systematic review of the evidence for traditional approaches to weight management through community pharmacy found that community pharmacy services were no more effective than private services and more expensive [19].

The aim of this paper is therefore to present the findings from an effective, long-term, private community pharmacy-led weight management service.

## 2. Materials and Methods

This is an evaluation of a private weight loss service provided from 2002 to 2019 by one community pharmacist (corresponding author) and a pharmacy assistant fully trained in weight management and use of the main dietary support programme, Lipotrim (Howard Foundation Research Ltd., Cambridge). The author named their service ‘Waistaway’. As an in-house private service evaluation, which had already been delivered, ethical approval was not sought.

### 2.1. Setting

The service was in four different community pharmacy clinics in Peterborough, Huntingdon, Wellingborough and Milton Keynes. Each participant was also supported by a pharmacy assistant fully trained in weight management and Lipotrim.

### 2.2. Recruitment

Individuals were recruited via written materials, website, social media, word of mouth and direct referral from other healthcare practitioners using a combination of bespoke media created by Waistaway and generic media sourced from Lipotrim. Free education sessions were offered and taken up by some healthcare professionals, including local GPs. Individuals were offered support and advice before, during and after following the programme, via a continually monitored mobile number and email. All written media, online, posters, flyers and leaflets, was designed with the aim of promoting the programme through motivational information regarding the health benefits of weight loss. No financial incentive, for example no discount or free offer, was used in recruiting individuals.

#### 2.2.1. Inclusion Criteria for VLCD

- Presenting with >6.35 kg of excess weight
- 18–75 years, unless under 18 years and fully through puberty or over 75, subject to clearance with Lipotrim helpline
- Initial BMI > 25, unless the clinical need exceeded the risk of non-inclusion

#### 2.2.2. Exclusion Criteria for VLCD

- BMI < 20
- Deemed likely to go to BMI < 20
- Medical conditions requiring postponement of dieting, such as pregnancy or breast feeding

#### 2.2.3. Criteria for Extra Vigilance by Pharmacist

Medical conditions requiring extra monitoring included hypertension and type 2 diabetes. Patients were directed to get their blood pressure (BP) checked each week for the first 2 weeks then fortnightly at their surgery. A patient reporting changes to their BP was actioned on by the pharmacist as referral to their GP for further assessment.

For those medicated for type 2 diabetes, GP co-operation was mandatory due to the need for medication review as a result of positive effects of weight loss. It is well recognized that the use of a VLCD shows near immediate improvement of blood glucose levels, highlighted recently in the DiRECT trial [20] which has led to the NHS imminently utilizing a VLCD to combat type 2 diabetes [21]. Similarly, this set of individuals were therefore only accepted on the programme if their GP had agreed to cease or dramatically lower their relevant medications in order to prevent hypogycaemia. Blood pressure and blood glucose data were not collected routinely for either of these cohorts since the service was not originally designed to capture such results. There are plans to capture these data in future, which we plan to evaluate in further studies.

### 2.3. Initial Consultation

The initial consultation involved identification of participant aims with respect to weight loss, past dieting history, medical conditions (if any) using a standardised medical questionnaire and discussion of any concerns they had resulting from agreeing an action plan. The pharmacist oversaw each patient’s acceptance on the programme if the pharmacy assistant carried out the initial consultation.

### 2.4. Intervention

Participants were required to attend a weekly weight check and monitoring appointment. VLCD was supplied on a weekly basis to maximise compliance and maintain adequate and necessary monitoring.

The weight loss approaches were divided up into two options, both available to each participant;

- Flexible plan for those identifying a desire to lose less than 6.35 kg, all other participants unwilling to use or unable to remain on the Sure plan (Tier 1 and 2 of the NICE guidelines)
- Sure plan for those with >6.35 kg weight loss need (Tier 3 of the NICE guidelines)

Any participants prescribed insulin (or other hypoglycaemic medications), or certain recognised medications such as warfarin or lithium, were restricted to the Flexible plan only, exceptions occasionally with GP cooperation only.

### 2.5. Flexible Plan

Consisted of the following weight loss aids to lose weight or to help maintain weight post weight loss;

- Food diary assessment: Completion of a food and drink diary for review and pharmacist advice on any obvious nutritional errors (cost to dieter: £3).
- Portion control: Introduction to the “Healthy Portion Plate”(Mariannes Plate, Hampshire) [22] or “The Diet Plate™”(Perfect Portion Control Ltd, Stockport) [23] as a possible one-off purchase (cost to dieter: £10 and £20 respectively) to help control the intake of calories and to allow correct portioning of the food groups, in line with the Eat-Well plate guidance [24] by the NHS.
- Partial Food Replacement (PFR): Nutritionally enriched formula foods (Lipotrim Maintenance range [25] and/or Ultralife SlimShake range (Company Dissolved, Sheffield) [26] were used to enable participants to supplement or replace conventional foods with a significantly reduced calorie intake without compromising their nutritional intake or satiety (cost to dieter, £2 per partial food replacement).

### 2.6. Sure Plan

Participants wanting to lose more than 6.35 kg and with no excluding criteria, such as medication or co-morbidities, were offered a Very Low Calorie Diet (VLCD), also known as a Very Low Energy Diet (VLED), option using Lipotrim’s nutritionally complete formula foods and protocols. On this total diet replacement (TDR) programme, participants are required to stop their intake of all conventional food immediately on the day they started the weight loss phase. Women immediately replaced their conventional food with three nutritionally complete formula foods a day (delivering 417.8 calories, 43 g carbohydrate, 5.8 g fat and 45 g protein per day) whilst men, who have higher nutrient requirements, instead replaced their food with 2 larger nutritionally complete formula foods per day (delivering 530.6 calories, 59.1 g carbohydrate, 7.4 g fat and 55.1 g protein per day). The remaining micronutrients were at levels of 100% or more Reference Daily Intake (RDI). Water, black tea and black coffee were the only drinks allowed to be used alongside the formula foods.

Prior to commencement of the VCLD, participants were required to watch an educational DVD and read the supplied comprehensive literature on how to effectively and safely implement the approach, including why and how to adhere to the programme requirements. This involved an explanation for the basis for the formula constituents, the basis behind the standardization of calorific intake for all participants, restrictions and possible side effects. It also introduced the concept of resuming conventional food intake correctly upon stopping the programme and how to manage long-term weight maintenance. This information was also discussed in their initial consultation. These consultations were completed by the pharmacist or overseen by the pharmacist if delivered by the pharmacy assistant in order to maintain medical control.

Advice from the pharmacist was provided, where appropriate, on how to manage their medical conditions whilst dieting. People with Type 2 diabetes on hypoglycaemic medications were signposted to their GP, using an advisory form, requesting the suspension of their medication for diabetes from day one of the diet. If the GP was not cooperative the diabetic participant was not permitted to participate on the Sure Plan. Since the VLCD usually normalised the blood sugars in just a few days, the continued use of hypoglycaemic medication could become problematic. Since it is highly likely significant weight loss will force diabetes remission, removing the need for continued diabetic medication, patients were referred back to their GP for a diabetes assessment on finishing.

For those prescribed antihypertensive therapy, blood pressure (BP) was additionally monitored at each time point. Participants prescribed antihypertensive medication were monitored weekly using a blood pressure monitor. Weight loss is well recognised to help control hypertension. At any point, if the pharmacist deemed a dose adjustment was required, they were referred to their GP whilst still maintaining diet compliance where appropriate. The participants were directed to seek a medication review by the GP after the discontinuation of the diet plan for whatever reason and returned to conventional foods.

Once the weight loss sequence was intended to be stopped, but prior to returning fully to conventional foods, participants followed a structured 7-day menu detailing a combination of formula foods and conventional foods called “refeeding”. This phase was to prevent weight gain from refeeding oedema. It is highly important to allow the participant to consume conventional foods without unnecessary discomfort or water retention after being carbohydrate restricted, for what is usually a significant amount of time.

The participant was provided with an audio CD, detailing the re-feeding phase, the rationale behind it and the principles of lifelong weight maintenance, including the use of the Waistaway Flex Plan. Since patients were at liberty to stop the diet at any time, the weight maintenance advice in terms of calorific intake and specific goals were given on an individual level.

The service was entirely participant funded. An average cost to a dieter using the Sure Plan (Lipotrim) per week, over the 17 years study period, has been £40.50 for females (21 formulas) and £54 for males (14 formulas). The pharmacist and assistant’s time, written materials to guide the dieter on how to follow the programme, any monitoring costs and a full complement of formulas for that individual for the forthcoming week was included within this cost. The pharmacist and assistant(s) overseeing the pharmacy service were paid in their usual capacity working for the company and no payment was necessary from the NHS or other healthcare provider. 

### 2.7. Data Collection

The following information was recorded for all participants from 2002 to 29 October 2019:

- Gender
- Relevant co-morbidities
- Age (Years)
- Height (m)
- Diet sequence number
- Weight (kg) at each consultation
- Body Mass Index (kg/m^2^)

Ketones were not measured in the pharmacy, with dieters able to purchase ketostix for the personal measuring of their dietary-ketosis levels at home. We ensured each dieter understood that the Sure Plan induces a mild dietary ketosis and this should not be confused with a pathological keto-acidosis.

Data input was carried out by the Waistaway pharmacist or assistant and quality was monitored by the pharmacist in charge, as is common practice in community pharmacy services.

### 2.8. Data Analysis

Data analysis was largely descriptive with means used for normally distributed data and medians for data which, on visual inspection, was clearly not. Percentages were calculated for nominal data.

A dieting “sequence” was defined as one week or more follow-up whilst using the Sure Plan VLCD. Duration was at the behest of the individual. A new sequence was defined as a ‘distinct new period of dieting initiation’ using the professional judgement of the pharmacist. An acute period of non-compliance of up to a week or sporadic non-compliance over a number of weeks was included within the diet sequence, whereas an extended period of complete non-compliance of more than a week was likely to result in a new sequence being started. A dieter who reached their lowest allowed BMI (20 kg/m^2^) or who terminated the weight loss phase themselves and moved on to the refeeding phase always ended their diet sequence. We recorded their last current engagement with the service as the end of the particular sequence and recorded weight loss at that point. Whilst length of sequence may be associated with increased weight loss, this is at the behest of the patient and dependent on their individual targets. As individuals frequently returned for additional weight loss, there were more sequences than individuals accessing the service.

The demographics, e.g., BMI and gender for individuals accessing the service and for sequences, were described. The average weight loss and % weight loss was calculated per individual and used to determine proportions of sequences where >5% weight loss was seen.

The number of sequences undertaken by each individual was recorded and this was used to determine whether number of events was related to any change in service efficacy.

Data for individuals with diabetes or hypertension were analysed separately to identify whether these groups responded differently.

## 3. Results

In total, 1738 individuals registered an initial weight on starting the Waistaway’s Sure Plan with 1023 (59%) individuals returning to register at least one more weight after starting their first VLCD diet sequence. A total of 1875 diet sequences were recorded and showed a median (IQ) 22 days (14,49) duration of weight loss attendance using the Sure plan, with a range of 3 to 472 days.

There were 715 “unsuccessful” “sequence one potential dieters (those not returning for a second weight check), 702 (98%) of the 715 presented only for an initial weight, 11 (1.5%) patients gained weight due to non-compliance and 2 (0.5%) records were unusable due to data input errors. These 715 individuals were not always lost to the service as some have presented for a further dieting sequence. An example of this can be seen in Table 1 (BMI category 20 to 24.9 kg/m^2^) which shows no males completing their first sequence, but 1 male completing a later diet sequence. Additionally, it needs to be understood a participant entering a new sequence may well start in a lower BMI category after earlier weight loss.

Out of the 1023 successful first sequence dieters, 872 (85.2%) were female, showing similar ratios at all dieting sequences per initial BMI. Median (IQ) starting BMI (kg/m^2^) was 31.9 (28.6, 36.2). Table 1 provides a profile of those participants who accessed the service and how many diet sequences were undertaken, detailed further with respect to gender and initial Body Mass Index (BMI). It can be seen that the majority of participants accessing the service were either overweight or obese. A significant proportion, however, had a BMI of greater than 35, often super or morbidly obese.

Total weight lost, across all 1875 dieting sequences, was 14,330 kg. Overall, there was a range of 0% to 58% weight loss from initial weight. Included within the data set, 20 sequences achieved some success but amounted to <1% weight loss and 405 sequences showed weight losses of 3% or less. 11 people gained weight.

Table 2 provides a summary of the total number of participant diet sequences which achieved >5% weight loss, the recognised threshold for clinical benefit. Results are expressed in kg lost, percent of initial weight lost by initial BMI categories. Over 60% of participants across all BMI categories achieved greater than 5% weight loss with this rising at higher BMI categories.

Table 3 provides a summary of the total number of diet sequences that achieved >5% weight loss, the recognised threshold for clinical benefit. The greatest number of sequences undertaken by one patient was 17 and irrespective of attempt number (up until the numbers become small) the percentage of individuals with >5% weight loss is above 60%.

### 3.1. People with Type 2 Diabetes

There were 11 participants who presented with type 2 diabetes having a mean (sd) starting BMI of 36.5 (6.6) kg/m^2^ and ended with a mean BMI of 32.8 (6.3) kg/m^2^. The mean (sd) weight loss was 11.2 (2.6) kg, equating to a mean percent (sd) weight loss of 10.4% (2.7).

### 3.2. People with Hypertension

There were 47 participants who presented with medicated hypertension having a mean (sd) starting BMI of 35.6 (6.1) kg/m^2^ and ended with a mean BMI of 31.8 (5.5) kg/m^2^. The mean (sd) weight loss was 11.1 kg (7.3) equating to a mean percent weight loss of 10.6% (5.9).

There was 1 patient who reported having reduced their blood pressure medication during a dieting sequence and 9 patients reporting blood pressure medication cessation during the dieting sequence. These data were not collected for many of the first years of service provision and patient-reported.

No diet-related adverse reactions were recorded that necessitated the discontinuation of the programme, including no reports of allergic reaction to the formula foods.

### 3.3. Weight Maintenance Using the Flex Plan

Of the 1875 Sure Plan diet sequences, 555 sequences saw participants accepting the offer of weight Maintenance support using the Waistaway Flex Plan, after using the VLCD.

In this separate analysis of the follow-on data, 173 (31%) of these cases showed a second weight check post weight loss could not be made.

The remaining 382 sequences saw individuals presenting for an unspecified number of weeks showing a mean (sd) weight gain of only 1.4 kg (4.3) (range = 20.4 kg gain to a 11.6 kg loss).

This equates to a mean (sd) % weight gain of only 1.8% (4.6) (range 29% gain to 15% weight loss).

Length of maintenance attendance was a mean (sd) 132 days (179) days post weight-loss (range 4 to 1099 days).

## 4. Discussion

This long-term service evaluation from one community pharmacist demonstrated that on average more than 50 patients each year accessed the service, resulting in over 100 diet sequences. Females are more likely to access the service than males, although males who did access the service tended to be more overweight. The average weight loss per diet sequence was 7.6 kg and a weight loss greater than 5%, required for clinical significance, was seen in over 65% of diet sequences. Of those losing greater than 5% initial weight, the percentage weight loss was frequently significantly more with an average of over 10%. Furthermore, the % weight loss increased in line with the starting weight of the individual, which is impressive given the greater actual weight loss this requires. In total, 35% of patients completed more than one diet sequence with a small number returning more than ten times. We anticipated that most patients would only undertake one diet sequence because in most cases they achieved the desired weight loss. Those who returned for a second sequence or more was most often to lose some weight they had subsequently put back on. Where individuals selected to maintain their weight through the follow-on service this was shown to largely maintain weight loss which is what is necessary to prevent future weight-related diseases and reduce existing disease progression.

This is a long-term service evaluation with a large number of patients. The consistency within the results with respect to weight loss and % weight loss demonstrates the consistency within service provision. Without a control arm, however, it is not possible to determine what would have happened if the service was not available, although published results on self-treated obesity or traditional weight loss advice is usually inferior. Furthermore, the nature of a service evaluation means that if a patient does not return for follow-up their data cannot be collected and long-term effects of the intervention remains unknown at that point. With individuals having been shown to return for further diet sequences, even in the face of initial dieting failure, there is not always a loss of long-term data and something to consider for further investigation. As the service is paid for by the individual and not the state, it is important to consider attrition of this nature as being, perhaps, of less concern.

With a large number of different elements within the service, we do not know which were most effective and which had little or no effect. A detailed process evaluation would enable identification of those elements of the service which work and those which have minimal impact [27]. This type of information could be used to make the service more efficient.

The generalisability of these results is limited due to the service being delivered by one very experienced and motivated individual. The results are such that it is worth comparing the Waistaway experience, in relation to the many other community pharmacies who are offering Lipotrim, to test service reproducibility. These results compare very favourably with that achieved by other Lipotrim pharmacies. The pharmacist providing the service has received no additional training in behaviour change counselling methods such as motivational interviewing [28]. By incorporating this, the effectiveness of the service may have been further enhanced [29,30].

The Flex Plan has two distinctly different roles, one for weight loss and one for weight maintenance. Our data collection software is currently designed to only audit the data for those following the Sure Plan, and not the weight loss data for those following the Waistaway Flex programme, when used solely for weight loss purposes. Therefore, it has only been possible to report the findings of using the Flex plan for weight maintenance after using the Sure plan. The software is currently in development to incorporate this.

Evidence suggests that males and females have preferences for different weight loss interventions [31]. The fact that males participate at a rate of 15% is testimony to the acceptance of the pharmacy environment and accessibility for men. Researchers looking into the experiences of males using a more traditional diet approach, have found that men prefer services with minimum time commitment and those which allow ‘treats’ within the program [32] The findings of such research may part explain the benefits and disadvantages of this model for males. The value from the rapidity of the VLCD weight loss and a general lack of medical knowledge on the benefits of the dietary restrictions imposed within the model of care presented here need to be recognised in order to remove any barriers to males accessing this service.

The main criticism of weight reduction services which are fully funded by governments through healthcare providers such as community pharmacies is that they are more expensive to the national health service than services provided by private providers [19]. This paper describes a private service delivered through a community pharmacy, which is both acceptable, demonstrated through the numbers of individuals who return, and is making a significant contribution to weight reduction. This service, as currently provided, costs the government nothing and yet it is likely to prevent future healthcare costs. Consequently, promotion of such services via governments and healthcare providers, combined with incentives for community pharmacies to set-up and offer such services, may be appropriate. With further economic analysis, this could be shown to be considerably cheaper than the NHS paying for such service provision, would provide another access route for patients and would offer reassurance with respect to the safe provision of care to individuals with co-morbidities such are hypertension and type 2 diabetes.

The weight losses seen in the number of people with diabetes were commensurate with those seen in those without type 2 diabetes, which is interesting given that it is stated as fact that “rates of weight loss may be slower in participants with type 2 diabetes” [33] (p. 47).

With these observations in mind, and the necessity for participation resting on the GP agreeing to suspend diabetic medication on starting the programme, we are interested in the future collection and further examination of blood glucose levels of this cohort to prove the remission of type 2 diabetes.

Similarly, this study has introduced the desirability for the pharmacy, not the GPs, to take the role of routinely measuring blood pressure for medicated hypertensive participants who were equally as successful losing weight on the programme. Future BP data will enable us to quantify the number of participants requiring dose reductions or even cessation of their antihypertensive medication as their blood pressure improved as a consequence of weight loss.

Since real weight loss is not always benign, a pharmacist is of significant value in the judgement of the safe conditions for the weight loss which can be necessary at any time point. The large weight losses often recorded confirm the need for a healthcare professional to facilitate any appropriate medication dose adjustments or medication cessation by the dieters GP. Unfortunately, without full access to the dieters NHS medical records, data could not be collected and analysed in terms of dose reductions or medication cessation after referral from the pharmacist.

## 5. Conclusions

The results show that a community pharmacist providing a private weight management service using a proprietary very low calorie diet programme can effectively support ongoing, clinically significant weight losses in large numbers of individuals over many years. This service costs governments nothing and yet can make a highly valuable contribution to the prevention of future weight-related illnesses.

Waistaway has devoted much time and effort achieving these significant results. As with establishing any pharmacy service above the core requirements, the expenditure of a pharmacist’s time needs to be taken into consideration. This, being a patient-funded service, allows any pharmacy wishing to follow suit to control the customer numbers to which they offer support according to their business capabilities.

With the availability of a healthcare professional and fully trained staff who can monitor outcomes for people with diseases affected by weight loss, it would seem appropriate for such services to be actively promoted by governments and incentive schemes provided to encourage more community pharmacies to set up such services.

## Figures and Tables

**Table 1:** Summary of participants accessing the service and the number of diet sequences by these participants.

BMI	No. Individuals Accessing the Service	No. (% per BMI Category) Males Accessing the Service	No. (% per BMI Category) Females Accessing the Service	No. (%per BMI Category) Individual Participants Completing 1st Diet Sequence	No. (%per BMI Category) Males Completing 1st Diet Sequence	No. (%per BMI Category) Females Completing 1st Diet Sequence	Total No. (% of Total No.) Diet Sequences	Total No. (% of Total No.) Diet Sequences Males	Total No. (% of Total No.) Diet Sequences Females
20–24.9	84	4 (5)	80 (95)	27 (32)	0 (0)	27 (3.1)	59 (3.1)	1 (0.4)	58 (3.6)
25–29.9	512	54 (11)	458 (89)	293 (57)	35 (23.2)	258 (29.6)	612 (32.6)	57 (21.9)	555 (34.4)
30–34.9	572	96 (17)	476 (83)	346 (60)	62 (41.1)	284 (32.6)	610 (32.5)	112 (43.1)	498 (30.8)
35–39.9	355	47 (13)	308 (87)	225 (63)	28 (18.5)	197 (22.6)	382 (20.4)	48 (18.5)	334 (20.7)
40–44.9	151	26 (17)	125 (83)	91 (60)	16 (10.6)	75 (8.6)	155 (8.3)	28 (10.8)	127 (7.9)
45–49.9	52	8 (15)	44 (85)	35 (67)	8 (5.3)	27 (3.1)	49 (2.6)	11 (4.2)	38 (2.4)
50–54.9	5	1 (20)	4 (80)	2 (40)	1 (0.7)	1 (0.1)	4 (0.2)	2 (0.8)	2 (0.1)
55–59.9	4	1 (25)	3 (75)	3 (75)	1 (0.7)	2 (0.2)	3 (0.2)	1 (0.4)	2 (0.1)
60–74.9	1	0	1 (100)	1 (100)	0 (0.0)	1 (0.1)	1 (0.1)	0 (0.0)	1 (0.1)
Bad data		0	2						
Total (%)	1738	237 (14)	1501 (86)	1023 (59)	151 (14.8)	872 (85.2)	1875 (100)	260 (13.8)	1615 (86.1)

**Table 2:** Losses per initial BMI category showing total number of successful diet sequences completed per BMI category and detailing the results of those achieving >5% weight loss and therefore medical benefit from the programme.

BMI Category	Total Number of Successful Diet Sequences Completed (All % losses)	No. (%) Participant Diet Sequences Achieving >5% Weight Loss from Initial Weight	Mean (sd) Weight Loss for Those Achieving >5% Weight Loss kg	Mean Weight Loss for Those Achieving >5% Weight Loss %	Weight Loss Range for Those Achieving >5% Weight Loss kg
20–24.9	59	36 (61.0)	5.2 (1.8)	7.6	2.8–11
25–29.9	612	408 (66.7)	6.8 (3.0)	8.8	2.8–18.1
30–34.9	610	407 (66.7)	9.5 (4.8)	10.4	3.5–32.1
35–39.9	382	254 (66.5)	12.8 (7.4)	12.5	4.3–40.9
40–44.9	155	112 (72.3)	15.7 (10.1)	13.3	4.8–49
45–49.9	49	38 (77.6)	18 (13.0)	13.8	6–61.1
50–54.9	4	3 (75.0)	45.1 (18.8)	31.7	23.4–56.5
55–59	3	3 (100.0)	48.2 (65.7)	23.7	10–124
60–75.9	1	0 (0)	0	0	0
Total	1875	1261	10.1 (7.7)	10.7	2.8–124

**Table 3:** Number of diet sequences and mean weight loss expressed in % and kg for those dieters achieving medical benefit (>5% weight loss of initial) per diet sequence number.

Diet Sequence Number	Total No. Diet Sequences *	No. (%) Diet Sequences * Achieving >5% Weight Loss	Mean Weight Loss % for Diet Sequences Achieving >5% Weight Loss	Mean(sd) Weight Loss kg for Diet Sequences Achieving >5% Weight Loss
1st	1031	749 (72.6%)	9.3	8.6 (8.4)
2	364	200 (54.9%)	9.3	8.5 (6.9)
3	183	112 (61.2%)	9.1	8.4 (5.7)
4	109	77 (70.6%)	10.1	9.6 (6.1)
5	57	36 (63.2%)	9.3	8.5 (4.5)
6	40	29 (72.5%)	12.6	12.6 (9.9)
7	21	14 (66.6%)	11.4	11.1 (7.6)
8	17	10 (58.8%)	10.4	10.1 (7.8)
9	20	13 (65%)	9.9	9 (5.4)
10	13	8 (61.5%)	8.4	7.5 (4.9)
11	10	4 (40%)	9.3	8 (1.4)
12	3	2 (66.6%)	8	8.2 (3.2)
13	2	2 (100%)	5	4.5 (0.2)
14	3	3 (100%)	11.7	11.2 (2.9)
15	1	1 (100%)	5	4.8
17	1	1 (100%)	12	12.1
total	1875	1261 (67.3%)	10.7	10.1 (7.0)

* Since each individual can only partake once in each diet sequence by definition, the description of No. diet sequences can also equivalently be described as Total Number of patients attempting each diet sequence. Note: the overall total number of dieting sequences (1875) does not reflect the total number of patients accessing the service.
